# Supplementary material for: The role of sociodemographic and psychosocial variables in early childhood development: A secondary data analysis of the 2014 and 2019 Multiple Indicator Cluster Surveys in the Dominican Republic
Source: PLOS Glob Public Health. 2022 Jul 19;2(7):e0000465. doi: 10.1371/journal.pgph.0000465 (PMC10021185; doi:10.1371/journal.pgph.0000465)
Supplement: S1 File — (DOCX) [file pgph.0000465.s001.docx]

**El papel de las variables sociodemográficas y psicosociales en el desarrollo de la primera infancia: Análisis de datos secundarios de las Encuestas de Indicadores Múltiples por Conglomerados de 2014 y 2019 en la República Dominicana**

**Resumen**

En la República Dominicana se ha establecido la asociación entre los factores sociodemográficos (como pobreza, falta de escolaridad materna y ser varón al nacer) y el retraso en el desarrollo infantil y los malos resultados educativos. Sin embargo, aún se desconocen los factores moderadores presentes o introducidos en las familias para amortiguar los efectos de dichos factores en el desarrollo infantil temprano. Realizamos un análisis secundario de las Encuestas de Indicadores Múltiples por Conglomerados de 2014 y 2019 para la República Dominicana, una encuesta nacional de hogares centrada en la salud y el desarrollo maternoinfantil. El primer objetivo de nuestro estudio fue confirmar la relación bien estudiada entre la posición socioeconómica, las prácticas de crianza y el desarrollo infantil. El segundo objetivo fue determinar si un modelo sociodemográfico predecía el desarrollo de la primera infancia. El tercer objetivo fue determinar si un modelo psicosocial (que incluye las prácticas familiares de crianza, la disciplina y la estimulación de la primera infancia) predecía el desarrollo de la primera infancia más allá del modelo sociodemográfico. El cuarto objetivo fue explorar la asociación entre las creencias de las madres sobre el castigo físico y las variables de desarrollo infantil y psicosociales. Encontramos que ambos modelos predijeron significativamente el desarrollo infantil, pero que el modelo psicosocial explicó más la varianza que el modelo sociodemográfico (6,3% en 2014 y 4,4% en 2019). Los predictores sociodemográficos más relevantes fueron la posición socioeconómica (que explicó el 21,6% de la varianza del desarrollo infantil en 2014 y 18,6% en 2019) y la educación de la madre (13,9% en 2014 y 14,1% en 2019). Los predictores psicosociales más destacados del desarrollo de la primera infancia fueron: la disciplina negativa, el número de libros para niños en el hogar, las actividades estimulantes en el hogar y la asistencia a un programa de educación de la primera infancia. Los pesos predictivos de las variables independientes fueron similares en ambos años. Estos resultados tienen múltiples implicaciones para los programas sociales que buscan mejorar el potencial de desarrollo de los niños y las niñas en contextos de pobreza. Aunque los resultados muestran el efecto protector de los factores psicosociales, la intervención sostenible y a gran escala no debe limitarse solo a efectos amortiguadores, sino a resolver realmente el problema subyacente que es que la pobreza impide que los niños y las niñas alcancen su potencial de desarrollo y les expone a un mayor riesgo de sufrir enfermedades crónicas a lo largo de su vida.
