# Supplementary material for: The role of sociodemographic and psychosocial variables in early childhood development: A secondary data analysis of the 2014 and 2019 Multiple Indicator Cluster Surveys in the Dominican Republic
Source: PLOS Glob Public Health. 2022 Jul 19;2(7):e0000465. doi: 10.1371/journal.pgph.0000465 (PMC10021185; doi:10.1371/journal.pgph.0000465)
Supplement: Attachment — Submitted filename: 220414 Response to reviewers.pdf [file pgph.0000465.s002.pdf]

Tulane University
School of Public Health and Tropical Medicine

April 14, 2022

Dear Dr. Biplab Kumar Datta,

We are pleased to submit the revised version of the manuscript titled "The role of sociodemographic and psychosocial variables in early childhood development: A secondary data analysis of the 2014 and 2019 Multiple Indicator Cluster Surveys in the Dominican Republic." As discussed with you on April 4th, we have included data from the 2019 database, which recently became available. We are thankful to you and the reviewers.

As you requested, we have provided separate figure files, we have included an Ethics Statement, and we have included the list of legends. We have also re-analyzed the data to account for sample weights.

In response to Reviewer 1:

- We reorganized the literature review by adding sub-sections.
- We have addressed the concern on confounding and omitted variables in the limitation section.
- We have specified the outliers in the methods section.
- Regarding the assumptions of linearity, normality, and homoscedasticity, we have added that the variance inflation factor statistics scored below 10 for all the variables, confirming that there was no multicollinearity.

In response to Reviewer 2:

- We reorganized the literature review. Although home visit programs were not the purpose of the study, the variable is somewhat measured in the psychosocial variables. We understand that the introduction reads better now since we included sub-headings that explore the factors that negatively impact ECD and the factors that positively impact ECD.
- We agree that ECD is not only a global health concern. We have reworded the sentence.
- We corrected the statistics about 249 million children not achieving full development and established the relationship between poverty and the rest of the variables (inequality, violence, stress, deficient care, lack of education and opportunities) in the section "Factors that negatively affect ECD."
- We have reviewed the study objectives.
- We agree that both sociodemographic and psychosocial variables together explain better the outcome and we have clarified the interpretation in the discussion section.
- We have included an additional description of the modeling steps in the methods section. Since we used the "Enter" method, all variables were kept in the model. All of them reached statistical significance. We have also included a figure presenting the hierarchical model—great suggestion, thank you!
- We have included additional descriptions of the modeling steps in the methods section.
- We included additional research questions in the introduction to account for the analysis presented in the results. In addition, we explained the analyses in the methods section and we moved Chronbach alphas to the method section.

Thank you for your consideration.

Sincerely,

Arachu Castro

Arachu Castro, PhD, MPH
Samuel Z. Stone Chair of Public Health in Latin America

Collaborative group for Health Equity in Latin America (CHELA), Department of Global Community Health and Behavioral Sciences
1440 Canal Street, Suite 2220, Mail Code #8331, New Orleans, LA 70112, USA, acastro1@tulane.edu
