# Supplementary material for: The role of sociodemographic and psychosocial variables in early childhood development: A secondary data analysis of the 2014 and 2019 Multiple Indicator Cluster Surveys in the Dominican Republic
Source: PLOS Glob Public Health. 2022 Jul 19;2(7):e0000465. doi: 10.1371/journal.pgph.0000465 (PMC10021185; doi:10.1371/journal.pgph.0000465)
Supplement: Attachment — Submitted filename: 220621 Response to editor and reviewers.pdf [file pgph.0000465.s003.pdf]

Tulane University
School of Public Health
and Tropical Medicine

June 21, 2022

Dear Dr. Biplab Kumar Datta,

We are pleased to submit the revised version of the manuscript titled "The role of sociodemographic and psychosocial variables in early childhood development: A secondary data analysis of the 2014 and 2019 Multiple Indicator Cluster Surveys in the Dominican Republic." We are thankful to you and the reviewers for helping us improve our manuscript.

As you requested, we have addressed all the reviewers' comments:

1. In response to reviewer-1's comment, the VIF statistics were provided. The VIF takes care of the multicollinearity issue, not heteroskedasticity. Please respond to the reviewer's comment on how the homoskedasticity assumption was met.
    - Done, lines 373-375. We used scatterplot of standardized residuals.

2. Please correct the typo in pg.3 lines 66-67. Please consider dividing the long sentence in lines 64-69 into two or more short sentences.
    - Done, lines 66-68.

3. In the Background section – 4 aims were mentioned. In the methods section, it's hard to follow how these aims were addressed. Please clearly outline what statistical analyses were performed to address each of the 4 aims. For example, for aim-1 following statistical analyses were performed ….. ; for aim-2 following statistical analyses were performed …
    - Done, lines 194-230.

4. Include a sentence stating whether the correlation between ECD and family's socioeconomic position in 2014 (i.e., 0.29) was different from that (i.e., 0.23) in 2019.
    - Done, lines 266-269. We provided observed z scores to test for differences between the magnitudes of the correlations.

5. The Pearson's correlation results presented in pg.12-13 are very difficult to follow. Please present the results in a table and describe the key findings in the text. In the table also indicate whether the correlation coefficients in 2014 were different from those in 2019.
    - Done in the text, lines 291-362, and Table 3 added, lines 364-367. We included household weight + z score.

    In the methods section state what tests were conducted to determine the differences in the correlation coefficients across two survey periods. Please indicate if the correlation analyses account for MICS survey weights. If not, provide justifications.
    - Done, lines 196-199.

6. The sentence "Descriptive statistics are displayed in Table 2." In pg.13 line 240 is redundant. It was already mentioned in pg.11 lines 212-213. Please drop one.
    - Done, line 362.

7. There are some redundancies in mentioning of the Pearson's correlation test and presentation of results in pg.11 lines 209-212 and pg.12-13 lines 223-228. Please rearrange the text to effectively communicate your findings.
    - We fixed this when addressing point 5.

Collaborative group for Health Equity in Latin America (CHELA), Department of Global Community Health and Behavioral Sciences
1440 Canal Street, Suite 2220, Mail Code #8331, New Orleans, LA 70112, USA, acastro1@tulane.edu

8. Remove the methodology of two-step hierarchical regression from the results section (pg.14 lines 249-257) in the methods section under respective aim.
    - Done, line 370.

9. What is beta in Table 3? Clearly state in the table foot note.
    - Done, line 418, now Table 4.

10. In the methods section clearly state how Figure 2 was created.
    - Done, lines 208-210.

11. In the footnote of table 4 clearly state what are F and (eta)2.
    - Done, line 510, now Table 5.

12. Compare whether the development scores in 2014 were statistically different from those in 2019.
    - We fixed this when addressing point 4.

13. In the discussion section, discuss the results by the 4 specific aims. It will help the readers to understand the important contribution of your work.
    - Done throughout the discussion.

14. Also please discuss how the estimates of 2014 were different (or not different) from that of 2019.
    - Done throughout the discussion for each aim.

Thank you for your consideration.

Sincerely,

Arachu Castro, PhD, MPH
Samuel Z. Stone Chair of Public Health in Latin America

Collaborative group for Health Equity in Latin America (CHELA), Department of Global Community Health and Behavioral Sciences
1440 Canal Street, Suite 2220, Mail Code #8331, New Orleans, LA 70112, USA, acastro1@tulane.edu
